# In Vitro Anti-Aging Effects of Yeast/Rice Fermentation Filtrate Combined with Sialic Acid in Cosmetic Applications

**DOI:** 10.3390/antiox14101184

**Published:** 2025-09-28

**Authors:** Fan Yang, Mingxuan Li, Yao Zuo, Lei Zhang, Jinyong Wu, Zhi Liu, Hua Wang

**Affiliations:** 1 Department of Biotechnology, College of Life Science and Technology, Huazhong University of Science and Technology, Wuhan 430074, China; 2 Research & Development Center, Mageline Biology Tech Co., Ltd., Wuhan 430206, China; 3 Research and Development Center, Wuhan CASOV Green Biotech Co., Ltd., Wuhan 430071, China; 4 Institute of Plasma Physics, Hefei Institutes of Physical Science, Chinese Academy of Sciences, Hefei 230031, China

**Keywords:** rice fermentation filtrate, sialic acid, anti-aging, antioxidation, anti-inflammation, extracellular matrix, collagen

## Abstract

Oxidative stress and chronic inflammation are major contributors to skin aging, promoting collagen degradation and impairing dermal structure. These factors stimulate the expression of matrix metalloproteinases, accelerating collagen breakdown and leading to wrinkles, sagging, and loss of elasticity. Given the key role of collagen in maintaining skin firmness and integrity, strategies that enhance collagen synthesis are essential for anti-aging interventions. This study investigated the combined effects of Yeast/Rice Fermentation Filtrate (RFF) and sialic acid (SA) on collagen production, antioxidation, and anti-inflammation, as well as their underlying mechanisms. The combination of RFF and SA significantly increased collagen genes transcription and mRNA stability, thereby enhancing collagen accumulation in the extracellular matrix. RNA-seq analysis revealed that RFF and SA modulate genes involved in the IL-17 signaling pathway. Mechanistically, RFF enhanced collagen mRNA stability by regulating HuR, while SA promoted collagen gene transcription via the TGF-β/Smad pathway. Moreover, the combination reduced the expression of inflammatory markers (IL-1β, IL-6, IL-8, PGE2, and NO) and improved cellular resistance to oxidative and inflammatory stress. These findings support the application of RFF and SA in anti-aging cosmetics and propose a novel strategy to enhance collagen production by simultaneously upregulating gene expression and stabilizing collagen mRNA.

## 1. Introduction

Skin aging is a complex, multifactorial process characterized by the appearance of fine lines, wrinkles, reduced skin elasticity, and diminished firmness [1]. Key factors, including ultraviolet (UV) radiation, oxidative stress, and inflammation, accelerate this process [2]. Collagen, the principal structural protein in the extracellular matrix (ECM), is essential for maintaining skin elasticity and firmness [3]. Type I collagen, constituting 80%~90% of the total collagen content, is the predominant form, followed by Type III (8%~12%) and Type V (~5%) [4]. As skin ages, the accumulation of reactive oxygen species (ROS) triggers the activation of matrix metalloproteinases (MMPs), leading to collagen degradation. These processes disrupt ECM homeostasis, reducing collagen levels and causing fibroblast dysfunction, leading to an aged phenotype with decreased ECM synthesis and increased matrix-degrading enzymes [5]. This imbalance of ECM homeostasis further drives collagen fibril fragmentation in a self-perpetuating cycle [6].

The TGF-β/Smad signaling pathway is well-established as the primary regulator of collagen synthesis [7,8,9]. However, the mechanism underlying post-transcriptional regulation of collagen genes remains unclear. The IL-17 signaling pathway plays a pivotal role in immune-mediated inflammatory responses and host defense [10,11]. IL-17A, the key cytokine in this pathway, activates downstream cascades via its receptor, leading to the production of pro-inflammatory cytokines and chemokines [12]. Emerging evidence indicates RNA-binding proteins (RBPs), such as HuR, act as critical modulators of IL-17-related transcripts by modulating their stability and translation, thereby fine-tuning inflammatory responses [13,14]. Within cells, RNAs associate with RBPs to form ribonucleoprotein complexes (RNPs), which govern RNA stability, localization, and function [15]. Notably, HuR enhances collagen gene expression by binding to the 3′ untranslated region (3′UTR) of collagen mRNAs, increasing their stability [16,17]. Thus, HuR-mediated stabilization of collagen transcripts may represent a key post-transcriptional mechanism regulating collagen production, offering potential targets for skin regeneration and anti-aging interventions.

Yeast/Rice Fermentation Filtrate (RFF) is a fermentation-derived extract rich in amino acids, organic acids, peptides, polysaccharides, polyphenols and vitamins, which collectively contribute to its diverse skincare benefits [18]. Research has shown that RFF exhibits moisturizing, skin barrier-reinforcing, antioxidant, anti-inflammatory, brightening, and anti-aging properties [19]. Our recent study also demonstrated that an 8-week application of an RFF-containing face cream significantly reduced wrinkle area and length around the eyes while enhancing skin firmness and smoothness [20]. These findings highlight the potential of RFF as a valuable active ingredient in anti-aging skincare formulations.

N-Acetylneuraminic acid, or sialic acid (SA), is widely distributed in vertebrates, especially in the brain, cerebrospinal fluid, and mucous secretions [21]. It is involved in key biological processes such as cell signaling, adhesion, and immune regulation [22,23]. In cosmetics, SA is valued for its moisturizing effect, enhancing skin hydration by attracting water molecules [24]. Its anti-aging properties stem from stimulating collagen synthesis, reducing fine lines, and providing antioxidant protection against oxidative damage. Furthermore, SA contributes to skin barrier repair by maintaining stratum corneum integrity and improving resistance to environmental stressors.

Previous studies have demonstrated that the combination of RFF and SA effectively promotes ECM components [25]; however, their optimal ratio, combined effects, and underlying mechanisms remain insufficiently explored. In this study, we investigated the combined anti-aging effects of RFF and SA, focusing on their dual regulatory roles at both transcriptional and post-transcriptional levels. Specifically, RFF enhances collagen mRNA stability by modulating HuR within the IL-17 signaling pathway, while SA promotes collagen gene transcription via the TGF-β signaling pathway. Furthermore, the combination significantly suppresses the expression of inflammatory mediators, such as IL-1β, IL-6, IL-8, PGE2, and NO, and enhances cellular resilience against oxidative and inflammatory stress. These findings provide novel insights into collagen post-transcriptional regulation and suggest promising strategies for skin anti-aging interventions.

## 2. Materials and Methods

### 2.1. Materials

Rice Fermentation Filtrate (RFF) was provided by Mageline Biology Tech Co., Ltd. (Hubei, China). Sialic acid (SA, purity ≥ 99%) was purchased from Wuhan CASOV Green Biotechnology Co., Ltd. (Wuhan, China).

### 2.2. Cell Culture

Human fibroblast cell (HFF-1 cells) [American Type Culture Collection (ATCC), CRL-3216] were cultured in Dulbecco’s modified Eagle’s medium (DMEM) supplemented with 10% fetal bovine serum (FBS; catalog no. S711-001, LONSERA). All cells were cultured at 37 °C with 5% CO_2_.

HFF cells were treated with SA and RFF at concentrations of 0.05%, 0.1%, 0.2%, 0.3%, 0.4%, 0.5%, 0.6%, 0.7%, and 0.8%, either individually or in combination at half concentrations (e.g., 0.5% SA, 0.5% RFF, or 0.25% SA + 0.25% RFF). Untreated HFF cells were used as the control group.

### 2.3. RNA Immunoprecipitation Assay

RNA immunoprecipitation (RIP) assays were conducted according to previously established protocols [26]. Specifically, human cell samples were supplemented with 1 × 10^6^ to 2 × 10^6^ HFF-1 cells, followed by fixation in 1% paraformaldehyde in PBS for 10 min. The fixation reaction was then quenched with 0.125 M glycine for 5 min. The fixed cells were subsequently subjected to nuclear isolation and chromatin shearing using the Diagenode Bioruptor Plus (Seraing, Belgium), operating in high-power mode for 25 cycles (sonication cycle: 30 s on, 30 s off). For immunoprecipitation, 5 μg of specific antibodies and 15 μL of pre-blocked protein A/G magnetic beads (Santa Cruz Biotechnology-CN, Shanghai, China) were used. After three washes with wash buffer [50 mM Hepes-KOH (pH 7.5), 300 mM LiCl, 1 mM EDTA, 1.0% NP-40, and 0.7% Na-deoxycholate], the immunoprecipitated DNA was eluted and reverse cross-linked using 200 μL of elution buffer [50 mM Tris-HCl (pH 8.0), 10 mM EDTA, 1.0% SDS, and proteinase K (200 μg/mL)] by incubation at 55 °C overnight. Finally, the DNA was purified through phenol-chloroform extraction followed by ethanol precipitation.

### 2.4. Transcriptomic Analysis

Total RNA was extracted using the RNeasy Kit (Qiagen, Wuhan, China), and its quality was assessed with an Agilent Bioanalyzer 2100 (Agilent, Santa Clara, CA, USA). Subsequently, mRNA was isolated using the KAPA mRNA Capture Kit (Roche, Basel, Switzerland), and cDNA libraries were constructed using the KAPA RNA Hyper Prep Kit (Roche) at Metware (Wuhan, China). Equivalent amounts of cDNA libraries from each sample were combined for sequencing on the Illumina HiSeq X platform (Illumina, San Diego, CA, USA), employing 150 bp paired-end sequencing. The sequencing depth achieved a median of 124.33766 million reads per sample, ranging from 96.444379 to 146.111230 million reads. The sequencing reads were aligned to the human reference genome GRCh38 using Hisat2 version 2.1.0 with default parameters. The aligned reads were then normalized to reads per million and converted into bigwig files for visualization. Genome annotations were obtained from the GRCh38 Ens_96 database and used to quantify gene expression with htseq-count version 0.13.5. Differential gene expression analysis was performed using R version 3.6.0 and the DESeq2 R 4.1.2 package. Differential splicing events were identified using rMATS, and significant events were filtered with the criteria: |ΔPSI| > 0.05 and false discovery rate (FDR) < 0.05.

### 2.5. Real-Time PCR for Collagen mRNA Detection

Total RNA was extracted from the transfected cells using TRIzol reagent (Thermo Fisher Scientific, Waltham, MA, USA) following the protocol provided by the manufacturer. The reverse transcription PCR was carried out using the cDNA Reverse Transcription Kit (Takara, Shiga, Japan). The primers used for detecting collagen mRNAs were designed based on previously published methods [26,27] and are listed in Table 1. Collagen mRNA levels were detected using a SYBR Green Master Mix Kit (Vazyme, Nanjing, China), and fold changes were calculated using the 2^−ΔΔCt^ method. GAPDH (human) were used as endogenous controls.

### 2.6. Luciferase Reporter Assay

For functional mechanistic analyses of SA and RFF, we predicted several Smad targets using the bioinformatics software Targetscan 7.1 and validated the candidates with the luciferase reporter assay. We generated 2 sets of vectors (pMIR-GLO Plasmid #212613, Merck, Darmstadt, Germany) containing the luciferase cDNA fused to the TGFB1, COL1A1, COL4A1 and CXCL8 3′-UTR (pMIR-GLO-COL1A1-3′UTR, pMIR-GLO-COL4A1-3′-UTR, pMIR-GLO-TGFB1-3′-UTR and pMIR-GLO-CXCL8-3′-UTR). HFF-1 cells were co-transfected with 0.4 μg of the reporter construct, 0.015 μg of the PGL3-basic (Plasmid #48743, Merck, Darmstadt, Germany) or pMIR-GLO control vector. After 48 h, the cells were harvested and assayed with the Dual Luciferase Assay Kit (Promega, Madison, WI, USA), according to the manufacturer’s instructions. All transfection assays were performed in triplicate.

### 2.7. ELISA

The protein levels of CXCL1, CXCL5, CXCL7, CXCL8, CXCL17, and MMP1 in the culture supernatants of treated HFF-1 cells were measured using enzyme-linked immunosorbent assay (ELISA) kits (CUSABIO, Wuhan, China) specific for each cytokine and MMP1 (CUSABIO, CSB-E17286m/ CSB-E08178h-IS/ CSB-E04562h/ CSB-RA582227A0HU/ CSB-EL006246HU/ CSB-E04672h). The assays were performed according to the manufacturer’s instructions. Briefly, culture supernatants were collected after 24 h of treatment and centrifuged at 1500 rpm for 10 min to remove cellular debris. The supernatants were then aliquoted and stored at −80 °C until analysis. ELISA plates were coated with capture antibodies specific to each target protein and incubated overnight at 4 °C. After washing with PBS containing 0.05% Tween-20 (PBST), the plates were blocked with 1% BSA in PBST for 2 h at room temperature. Supernatant samples were added to the wells and incubated for 2 h at room temperature. Detection antibodies were then added and incubated for an additional 1 h, followed by the addition of streptavidin-conjugated horseradish peroxidase (HRP) for 30 min. The substrate solution (TMB) was added, and the reaction was terminated by adding 2M sulfuric acid. The absorbance was measured at 450 nm using a microplate reader (BioTek Instruments, Winooski, VT, USA). The concentrations of each protein were determined by comparing the absorbance values to a standard curve generated from recombinant proteins of known concentrations.

### 2.8. Nucleocytoplasmic Separation

Nucleocytoplasmic separation was performed using the NE-PER Nuclear and Cytoplasmic Extraction Reagents (Thermo Fisher Scientific, Waltham, USA) according to the manufacturer’s instructions. Briefly, treated HFF-1 cells were washed twice with cold PBS and then scraped into cold homogenization buffer containing protease inhibitors. The cell lysate was centrifuged at 700× *g* for 5 min at 4 °C to pellet the nuclei. The supernatant, representing the cytoplasmic fraction, was carefully collected and stored at −80 °C. The nuclear pellet was resuspended in nuclear extraction buffer, vortexed intermittently for 15 min, and then centrifuged at 16,000× *g* for 5 min at 4 °C. The supernatant containing the nuclear proteins was collected and stored at −80 °C. Protein concentrations in both cytoplasmic and nuclear fractions were measured using the BCA Protein Assay Kit (Thermo Fisher Scientific). Equal amounts of protein (30 µg) from each fraction were separated by SDS-PAGE on a 10% polyacrylamide gel and transferred to a PVDF membrane (Merck, Darmstadt, Germany). The membrane was blocked with 5% non-fat milk in TBST (Tris-buffered saline with 0.1% Tween-20) for 1 h at room temperature. The membrane was then incubated overnight at 4 °C with primary antibodies against HuR (rabbit anti-HuR, 1:1000 dilution; Santa Cruz Biotechnology) and GAPDH (mouse anti-GAPDH, 1:5000 dilution; Merck, Darmstadt, Germany) or Lamin B1 (mouse anti-Lamin B1, 1:2000 dilution; Abcam, Cambridge, UK) as cytoplasmic and nuclear loading controls, respectively. After washing with TBST, the membrane was incubated with secondary antibodies (goat anti-rabbit IgG-HRP or goat anti-mouse IgG-HRP, 1:5000 dilution; Santa Cruz Biotechnology) for 1 h at room temperature. The protein bands were visualized using an enhanced chemiluminescence (ECL) detection system (Bio-Rad, Hercules, CA, USA) and quantified using ImageJ 1.8.0 software.

### 2.9. Western Blot

Whole-cell lysates were subjected to Western blot analysis. Rabbit polyclonal anti-COL1A1 antibody (6994-1; 1:1000) and anti-COL4A1 antibody (ab35962; 1:1000) that from Epitomics (Abcam, Cambridge, MA, USA), or a GAPDH antibody (60004-1-Ig, Proteintech, Rosemont, IL, USA; 1:100,000) were used as the primary antibodies and incubated in 1× TBST containing 5% BSA for 2 h at room temperature (RT) with continuous shaking. After washes with 1× TBST for 15 min at RT, the membranes were incubated with an HRP-conjugated goat anti-rabbit secondary Ab (Thermo Fisher Scientific, San Jose, CA, USA) for 1 h at RT. After a final washing step with 1× TBST for 15 min at RT, the immunoreactive bands were visualized by enhanced chemiluminescence (ECL) (Amersham Biosciences, Piscataway, NJ, USA) at indicated exposure time (“Exposure time for Western blot”).

### 2.10. Statistical Analysis

All data was presented as the means ± standard deviation (SD) of three experiments. Analysis was performed using Student’s *t*-test or ANOVA when it is appropriate. A *p*-value less than 0.05 was considered statistically significant.

## 3. Results

### 3.1. RFF–SA Combination Significantly Enhance Collagen Expression

**Figure 1:**
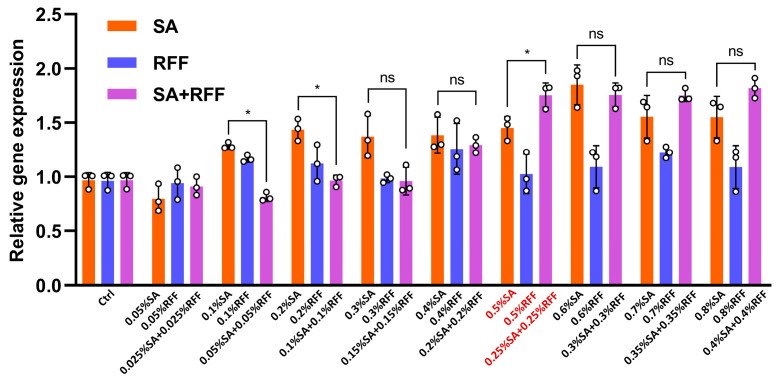
The RFF–SA combination significantly enhances collagen expression. mRNA levels of COL1A1 were quantified by real-time PCR in HFF-1 cells treated with SA, RFF, or their combination. Data are presented as mean ± SD from three independent experiments. * *p* < 0.05.

Type I collagen, which constitutes approximately 80–90% of total dermal collagen, plays a vital role in maintaining skin structure and elasticity, and is thus a key target in anti-aging strategies. To determine the optimal combined concentration of RFF and SA, HFF-1 cells were treated with SA and RFF at 0.05–0.8%, either individually (e.g., 0.05% SA or 0.05% RFF) or in combination at half the corresponding single-agent concentrations (e.g., 0.025% SA combined with 0.025% RFF), with untreated cells serving as the control. The expression of the COL1A1 gene in HFF-1 cells was quantified by RT-qPCR. As shown in Figure 1, COL1A1 expression increased significantly with rising concentrations of SA compared to the control group, whereas RFF alone induced only a modest increase. When the concentrations of SA and RFF in the combination group were below 0.25%, the combined treatment was less effective than the corresponding single-agent treatments at 0.5%. Notably, When the concentrations of SA and RFF in the combination group reached 0.25% or higher, the combination began to surpass the single-agent treatments in upregulating COL1A1. Specifically, 0.25% SA combined with 0.25% RFF further enhanced COL1A1 expression, showing a 20.8% increase compared to 0.5% SA alone. Further increases in concentration did not produce additional enhancement, suggesting a plateau effect. Therefore, 0.25% SA combined with 0.25% RFF was adopted as the optimal combination for subsequent experiments.

### 3.2. RFF–SA Combination Regulate Anti-Aging Genes via the IL-17 Signaling Pathway

**Figure 2:**
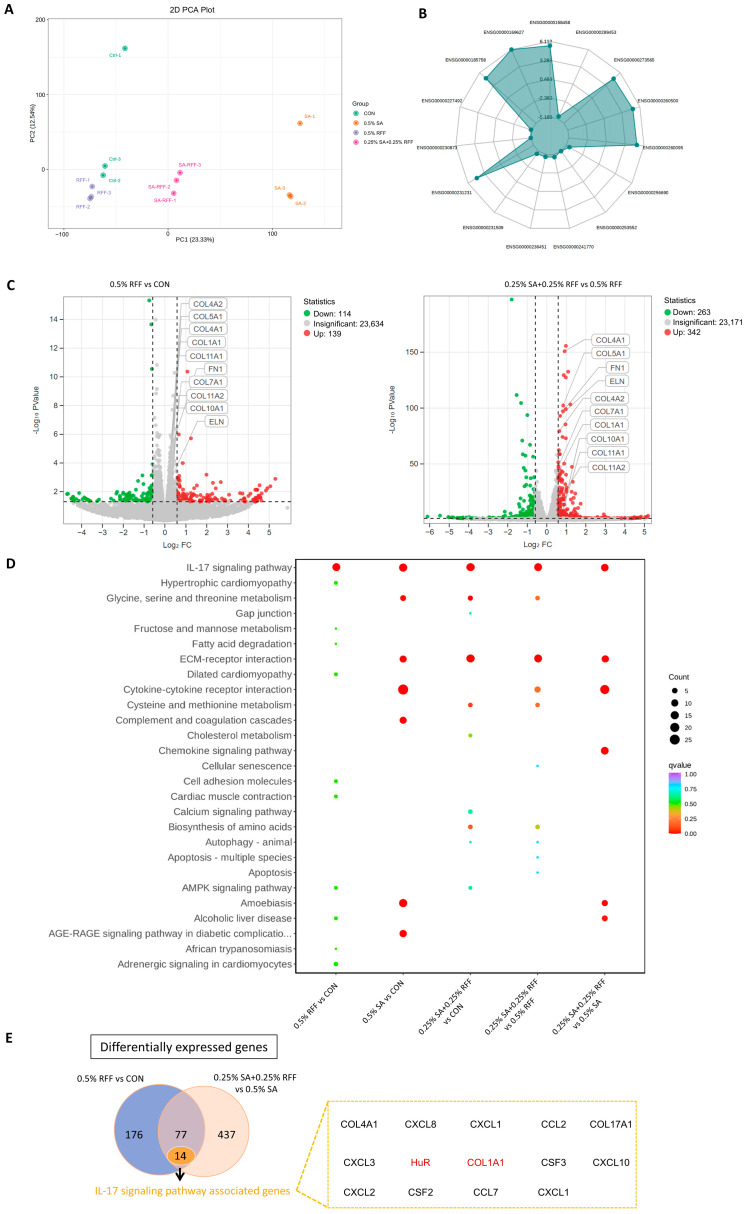
Regulation of anti-aging genes by the RFF–SA combination via the IL-17 signaling pathway. (**A**) Principal component analysis (PCA) comparing intra- and intergroup gene expression differences among control and cells treated with SA, RFF, or the RFF–SA combination. (**B**) Radar plot depicting 10 genes significantly upregulated in SA and RFF treatment groups relative to control. (**C**) Volcano plot illustrating differentially expressed genes in cells treated with the RFF–SA combination compared to the SA group; upregulated genes are shown in red, downregulated genes in green. (**D**) KEGG pathway enrichment analysis of differentially expressed genes in HFF-1 cells treated with the RFF–SA combination based on RNA-Seq data. (**E**) A Venn diagram showing the differentially expressed genes. Data are presented as mean ± SD from three independent experiments.

To further elucidate the molecular mechanisms underlying the combined effects of RFF and SA, we investigated their roles in the pro-collagen process using the previously identified optimal concentrations (0.25% SA combined with 0.25% RFF). HFF-1 were treated with 0.5% RFF, 0.5% SA, or a combination of 0.25% SA and 0.25% RFF (SA + RFF), with untreated cells serving as the control. After 24 h, total RNA was extracted and subjected to high-throughput RNA sequencing. Principal component analysis (PCA) of the RNA-seq data demonstrated good consistency among biological replicates (Figure 2A). Given previous reports identifying RFF and SA as pro-collagen agents [25], we next examined their influence on collagen-related gene expression pathways. A radar plot showing ten genes that were significantly differentially expressed in the RFF–SA Combination group compared to the control (Figure 2B). Furthermore, the combination treatment led to a notable upregulation of multiple collagen-related transcripts (log_2_FC > 0.5, q-value < 0.05) (Figure 2C). Interestingly, gene expression profiles varied in response to increasing SA concentrations, suggesting a dose-dependent regulatory effect.

Given the well-established role of the TGF-β/Smad signaling pathway in regulating collagen gene expression [28], we next performed KEGG analysis on differentially expressed genes in HFF-1 cells treated with the RFF–SA combination. Compared to the untreated control group, among the 636 genes identified (log_2_FC > 0.5, q-value < 0.05), significant enrichment was observed in the ECM–receptor interaction, cytokine–cytokine receptor interaction, and IL-17 signaling pathways (Figure 2D). Notably, the ECM–receptor interaction pathway, which is closely linked to TGF-β/Smad signaling [29], was prominently enriched. To further clarify RFF’s contribution to the combination treatment, we performed independent pathway enrichment analysis on genes regulated by RFF alone. The results indicated that RFF primarily modulated genes associated with the IL-17 signaling pathway within the combination group, highlighting a potential role for IL-17 signaling in mediating its combined effects with SA.

To further clarify the mechanism by which the combination of SA and RFF enhances COL1A1 gene expression, and considering that SA is known to regulate the TGF-β signaling pathway, we investigated the signaling pathways modulated by RFF alone or in combination with SA. A Venn diagram analysis comparing the RFF vs. control group with the SA + RFF vs. SA group identified 77 commonly differentially expressed genes, 14 of which were enriched in the IL-17 signaling pathway (Figure 2E). These findings suggest that the combination of SA and RFF regulates collagen expression through the IL-17 signaling pathway, thereby establishing a dual regulatory mechanism in conjunction with the TGF-β signaling pathway.

### 3.3. SA Strongly Enhances Collagen Gene Promoters Compared to RFF

**Figure 3:**
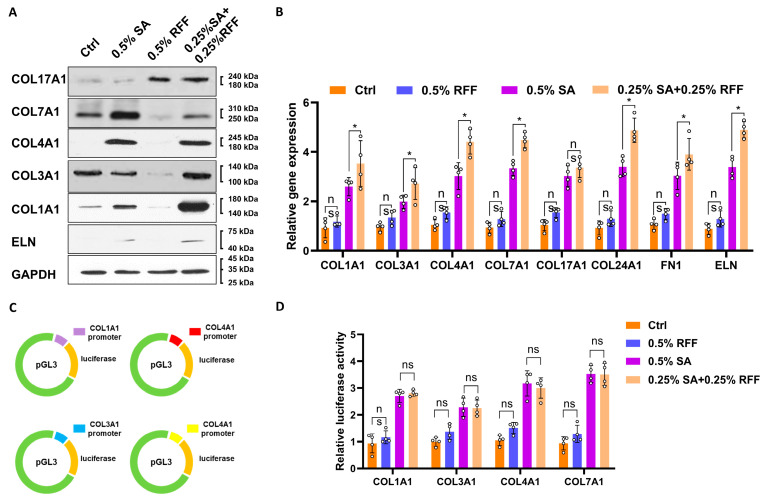
Effect of SA and RFF on Promoter Activities of Multiple Collagen Genes. (**A**) Western blot analysis of protein levels of COL1A1, COL3A1, COL4A1, COL7A1, COL17A1, and elastin (ELN) in HFF-1 cells treated with SA, RFF, or the combination for 24 h. (**B**) mRNA expression levels of COL1A1, COL3A1, COL4A1, COL7A1, COL17A1, COL24A1, fibronectin (FN1), and ELN in HFF-1 cells after treatment with SA, RFF, or the combination, measured by real-time PCR. (**C**) Schematic representation of dual fluorescent reporter constructs containing promoter regions of COL1A1 (purple), COL3A1 (blue), COL4A1 (red), and COL7A1 (yellow) cloned into the pGL3 vector. (**D**) Luciferase assay measuring promoter activities of COL1A1, COL3A1, COL4A1, and COL7A1 in HFF-1 cells treated with SA, RFF, or the combination. Data are presented as mean ± SD from three independent experiments. * *p* < 0.05.

HFF-1 cells were treated with 0.5% SA, 0.5% RFF, or a combination of 0.25% SA and 0.25% RFF, while untreated cells served as the control. The expression of key ECM genes was subsequently analyzed by RT-PCR. SA treatment resulted in a statistically significant upregulation of COL1A1, COL3A1, COL4A1, COL7A1, COL17A1, and COL24A1 gene expression, whereas RFF alone did not induce significant changes. In contrast, RFF did not induce a statistically significant increase. Notably, the combined treatment of RFF and SA significantly increased the expression of these ECM genes compared to the control (*p* < 0.05). This was further corroborated by Western blot results, which showed that the protein expression levels were consistent with the gene expression findings (Figure 3A,B). To demonstrate whether SA and RFF combination regulate collagen gene promoter, promoter fragments of COL1A1, COL3A1, COL4A1, and COL7A1 were cloned into the pGL3 vector (Figure 3C). Luciferase assays showed no significant enhancement of collagen promoter activity in the combined RFF and SA treatment group compared to SA alone (Figure 3D). Consequently, we focused on an alternative regulatory mechanism involving the IL-17 signaling pathway, specifically the modulation of mRNA stability by the RNA-binding protein HuR [30,31].

### 3.4. RFF–SA Combination Promotes HuR Nuclear Translocation

**Figure 4:**
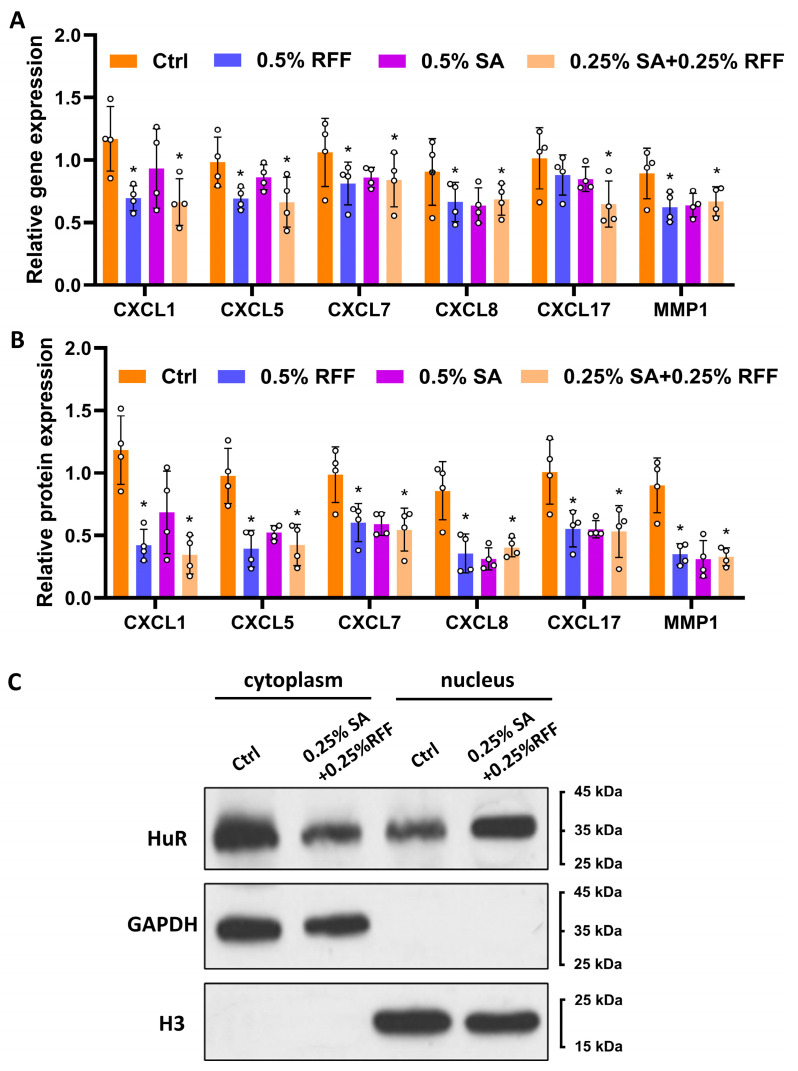
RFF–SA combination promotes nuclear translocation of HuR in fibroblasts. (**A**) mRNA expression levels of pro-inflammatory markers (CXCL1, CXCL5, CXCL7, CXCL8, CXCL17, and MMP1) were measured in HFF-1 cells treated with SA, RFF, or the combination of SA and RFF using real-time PCR. (**B**) Protein levels of the same markers were quantified by ELISA after 24 h treatment. (**C**) Cytoplasmic and nuclear fractions were isolated from treated HFF-1 cells, and HuR protein distribution was analyzed by Western blotting. Data are presented as mean ± SD from three independent experiments. * *p* < 0.05.

HFF-1 cells were treated with 0.5% SA, 0.5% RFF, or a combination of 0.25% SA and 0.25% RFF, with untreated cells serving as the control. RT-PCR was then performed to assess the expression of CXCL1, CXCL5, CXCL7, CXCL8, CXCL17 and MMP1, which are downstream targets of the IL-17 signaling pathway. The results showed that the expression of these genes was significantly inhibited by RFF and slightly inhibited by SA, while their combination did not show a greater inhibition than RFF alone (Figure 4A). This was consistent with the ELISA results, which demonstrated similar protein expression levels inhibited by RFF (Figure 4B). HuR is an important RNA-binding protein in the IL-17 pathway that binds to the 3′UTR domain of gene mRNAs, increasing their stability [32]. Thus, we treated HFF-1 cells with the combination of SA and RFF and examined HuR protein levels in the cytoplasm and nucleus using Western blot analysis after separating the cytoplasm and nucleus of the cells. Combined with RNA-seq results, this indicates that the combination of RFF and SA promotes the translocation of HuR proteins from the cytoplasm to the nucleus, with RFF playing a predominant role in this process (Figure 4C).

### 3.5. RFF–SA Combination Increase COL1A1 mRNA Stability

**Figure 5:**
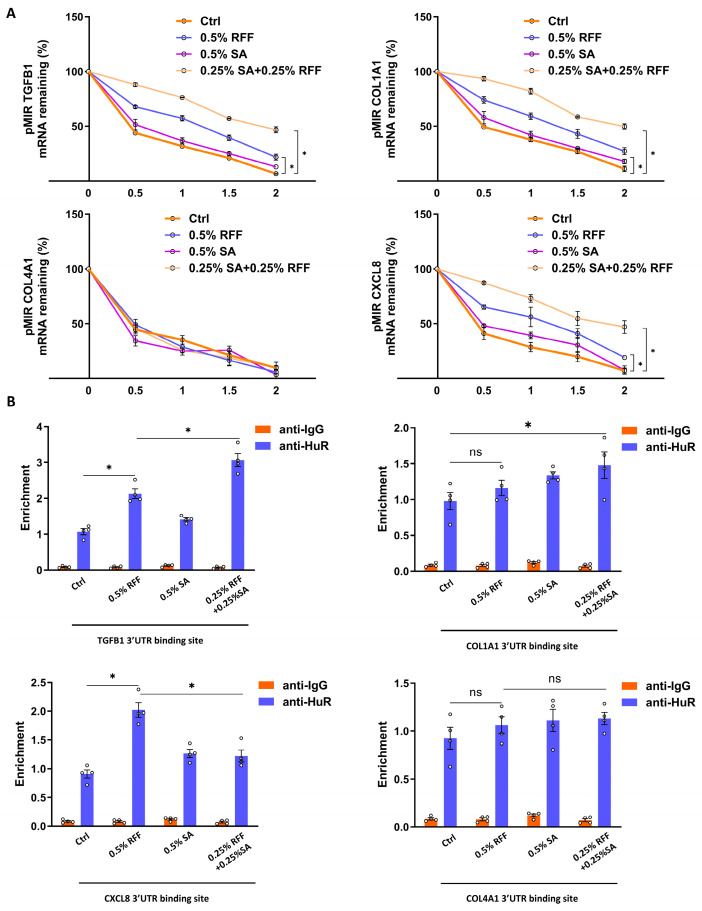
RFF–SA combination increases COL1A1 mRNA stability. (**A**) mRNA stability of COL1A1, COL4A1, TGFB1, and CXCL8 was assessed in HFF-1 cells treated with SA, RFF, or their combination. (**B**) RNA immunoprecipitation (RIP) was performed to evaluate the binding of HuR to COL1A1, COL4A1, TGFB1, and CXCL8 mRNAs in HFF-1 cells. Data are presented as mean ± SD from three independent experiments. * *p* < 0.05.

HFF-1 cells were first treated with the global methylation inhibitor 3-deazaadenosine (3-DAA), followed by treatment with 0.5% RFF, 0.5% SA, or a combination of 0.25% RFF and 0.25% SA. Untreated cells served as the control group. Subsequently, HFF-1 cells were transfected with reporter plasmids containing the 3′ untranslated regions (3′UTRs) of COL1A1, COL4A1, TGFB1, or CXCL8 (pMIR-GLO-COL1A1-3′UTR, pMIR-GLO-COL4A1-3′UTR, pMIR-GLO-TGFB1-3′UTR, and pMIR-GLO-CXCL8-3′UTR), and cultured for 24 h in the presence or absence of 3-DAA. Luciferase activity assays were then performed to assess mRNA stability. Compared with the control, both RFF alone and RFF combined with SA significantly enhanced luciferase mRNA stability (Figure 5A).

To further investigate whether RFF and SA regulate mRNA stability via HuR, RNA immunoprecipitation (RIP) assays were performed. Cell lysates were precipitated using magnetic beads conjugated with IgG or HuR antibodies to detect HuR binding to the COL1A1 3′UTR. The results demonstrated that both SA and RFF significantly promoted HuR binding to the COL1A1 3′UTR (Figure 5B). Together, these findings suggest that, unlike SA, RFF enhances collagen gene expression by stabilizing mRNA and inhibiting its degradation.

### 3.6. RFF–SA Combination Increase Skin Cell Resistance to Inflammatory Responses and Oxidation

**Figure 6:**
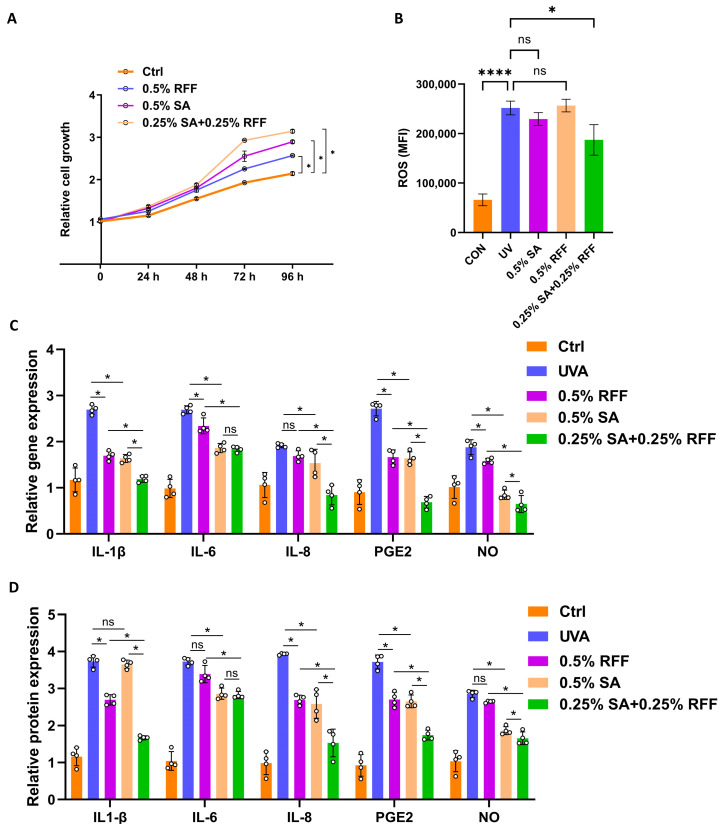
RFF–SA combination increases skin cell resistance to inflammatory responses and oxidative stress. (**A**) Cell proliferation of HFF-1 cells treated with SA, RFF, or RFF–SA combination was assessed using the CCK-8 assay. (**B**) Intracellular ROS levels were measured by flow cytometry. mRNA levels of IL-1β, IL-6, IL-8, PGE2, and NO in HaCaT cells after 24 h treatment with SA, RFF, or their combination, assessed by real-time PCR. (**C**) Gene expression levels of IL-1β, IL-6, IL-8, PGE2, and NO. (**D**) Protein levels of IL-1β, IL-6, IL-8, PGE2, and NO were quantified by ELISA. Data are presented as mean ± SD from three independent experiments. * *p* < 0.05; **** *p* < 0.0001.

The IL-17 signaling pathway plays a pivotal role in mediating cellular inflammatory and oxidative responses [33]. To evaluate the protective effects of RFF and SA, we first assessed cell proliferation using the CCK-8 assay. Cells treated with 0.5% SA, 0.5% RFF, or the combination of 0.25% RFF and 0.25% SA served as experimental groups, while untreated cells served as controls. HFF-1 cells treated with the combination of RFF and SA exhibited a significantly higher proliferation rate compared to either treatment alone or the control group (Figure 6A), with an increase of approximately 41.7% over the control at 96 h (*p* < 0.05). Next, intracellular reactive oxygen species (ROS) levels were measured by flow cytometry. The RFF-SA combination group exhibited a significantly greater reduction in ROS generation compared to the UVA group, as well as the groups treated with RFF or SA alone, with fluorescence intensity decreasing by approximately 25.7% (*p* < 0.05), indicating enhanced antioxidative capacity (Figure 6B).

To further evaluate the anti-inflammatory effects, we measured both mRNA and protein levels of key pro-inflammatory cytokines, including IL-1β, IL-6, IL-8, PGE2, and NO. Real-time PCR analysis demonstrated that treatment with SA, RFF, and particularly their combination significantly downregulated the expression of these genes. In the RFF-SA combination group, the mRNA level of IL-8 and the protein levels of IL-1β, IL-6, and IL-8 were reduced by 55.9%, 31.8%, and 68.8%, respectively, compared to the UVA group (*p* < 0.05; Figure 6C). In addition, PGE2 and NO levels were decreased by 74.6% and 76.0%, respectively. Consistent with the PCR results, ELISA analysis confirmed a corresponding reduction in the secretion levels of IL-1β, IL-6, IL-8, PGE2, and NO following treatment with the RFF-SA combination (*p* < 0.05; Figure 6D). Notably, the RFF-SA combination group exhibited markedly greater anti-inflammatory effects than either treatment alone (*p* < 0.05). These results indicate that the combined combination of RFF and SA enhances the resistance of skin fibroblasts to inflammatory and oxidative stress by promoting cell proliferation, reducing ROS accumulation, and downregulating inflammatory mediators at both the gene and protein levels.

## 4. Discussion

Skin aging is characterized by fine lines, wrinkles, laxity, and uneven pigmentation, which are accelerated by environmental factors such as UV radiation, oxidative stress, and chronic inflammation [34]. Collagen, a key structural protein responsible for skin elasticity and firmness, progressively degrades with age due to the accumulation of ROS and increased MMP activity [35]. In this study, we investigated the combined anti-aging effects of RFF and SA, with a specific focus on their roles in collagen production, oxidative defense, and inflammation modulation.

Our results demonstrate that the combination of 0.25% RFF and 0.25% SA significantly enhances collagen synthesis, achieving a 198% increase compared with 0.5% RFF alone and a 36% increase compared with 0.5% SA alone. RNA-seq and RT-qPCR analyses revealed distinct but complementary mechanisms underlying their combined action. Specifically, SA primarily enhances collagen gene transcription by increasing collagen promoter activity through the TGF-β signaling pathway, leading to greater accumulation of collagen mRNA and ultimately increased collagen content. However, due to inherent post-transcriptional regulation and mRNA instability, merely increasing transcription cannot fully prevent degradation, thereby limiting protein expression. Conversely, RFF modulates the RNA-binding protein HuR within the IL-17 signaling pathway. This not only elevates gene expression levels but also facilitates the translocation of HuR protein from the cytoplasm to the nucleus. RIP experiments confirmed that both RFF alone and the combination of SA and RFF enhance the binding of HuR protein to the 3′UTR domains of genes such as COL1A1. Together, these findings support a dual mechanism in which SA upregulates gene transcription while RFF stabilizes transcripts, resulting in amplified collagen production.

Moreover, our study indicates that this combined treatment significantly enhances collagen expression levels while enabling equivalent pro-collagen effects at lower concentrations. Transcriptomic analysis further revealed that the combination of SA and RFF enriched differentially expressed genes in the ECM–receptor interaction and cytokine–cytokine receptor interaction signaling pathways compared with the control group. In addition, both SA and RFF inherently possess anti-inflammatory effects, the combination markedly suppressed ROS and key pro-inflammatory mediators, including IL-1β, IL-6, IL-8, PGE2, and NO, while enhancing HaCaT keratinocyte resistance to oxidative stress and inflammatory stimuli. These results suggest that beyond collagen-related effects, RFF and SA exert broader cytoprotective and anti-inflammatory actions.

The observed combined effects of RFF and SA align with broader trends in dermatology, where combination therapies often outperform monotherapy by targeting multiple pathways. For instance, resveratrol combined with emodin has been reported to markedly enhance antioxidant activity [36]. Similarly, topical application of Vitreoscilla filiformis extract in combination with Vichy volcanic mineralizing water has been reported to reinforce the skin barrier, stimulate antimicrobial peptide production, and modulate immune defenses, thereby enhancing skin resilience [37]. In the context of pigmentation, High Mobility Group Box 1 (HMGB1) promotes melanocyte dendricity and melanosome transfer without affecting melanin synthesis, whereas sucrose laurate suppresses HMGB1 release, reduces dendricity, and limits melanosome transfer [38]. These complementary actions suggest a regulatory balance in pigmentation through coordinated modulation of keratinocyte–melanocyte signaling and melanosome transfer. These examples illustrate how combination therapies can achieve complementary effects by simultaneously modulating multiple biological processes, supporting the concept of multi-targeted interventions in skincare.

Beyond their effects on collagen metabolism, transcriptomic data indicate that RFF and SA also influence additional signaling pathways implicated in skin aging and repair, such as PI3K-AKT, TNF, and NF-κB pathways [39]. The PI3K-AKT pathway regulates cell growth, survival, and metabolism, and its activation is linked to cell proliferation and apoptosis inhibition. By modulating this pathway, RFF and SA could enhance skin cell vitality and delay aging through promoting cell survival and metabolic health. NF-κB, involved in immune and inflammatory responses, is often dysregulated in aging and skin conditions [40]. RFF and SA’s impact on this pathway suggest potential anti-inflammatory properties, protecting the skin from chronic inflammation. By regulating cell survival and inflammation, RFF and SA may also accelerate wound healing and improve skin repair. Moreover, their effects on the PI3K-AKT imply a capacity to delay cellular aging and possibly influence other age-related processes beyond collagen degradation. Further studies are warranted to explore the broader roles of RFF and SA in skin health and anti-aging, underscoring their potential as multifaceted dermatological agents.

While this study presents promising results, some limitations should be acknowledged. We only evaluated the in vitro efficacy in cultured fibroblasts, without in vivo validation. Future work should therefore include animal models or clinical studies to confirm translational relevance. Moreover, although previous studies have demonstrated favorable skin permeability of RFF and SA [25], it remains necessary to investigate their penetration and bioavailability in greater detail, as the delivery and stability of these actives can markedly influence biological activity. Future studies should therefore examine penetration at the cellular level, in 3D skin equivalents, and in human clinical settings. Additionally, the dose–response relationship and long-term safety warrant further evaluation.

In conclusion, we have demonstrated that the combination of RFF and SA jointly enhances collagen production through the IL-17 signaling pathway. RFF regulates the RNA-binding protein HuR, stabilizing collagen mRNA, while SA upregulates collagen gene transcription via the TGF-β pathway. The combination not only promotes collagen synthesis but also enhances skin resistance to inflammation and oxidative stress. Overall, our findings highlight the potential of RFF and SA as enhanced anti-aging agents, offering a novel strategy to boost collagen production and maintain skin vitality.

## Figures and Tables

**Table 1:** Primers for real-time PCR and RIP-PCR.

Gene Symbol (Accession Number)	Sense Primer (5′–3′)	Antisense Primer (5′–3′)
COL1A1 (NM_000088.4)	AAGGTGTTGTGCGATGACG	TGGTCGGTGGGTGACTCTG
COL3A1 (NM_000090.4)	CCCGTATTATGGAGATGAAC	TCAGGACTAATGAGGCTTTCT
COL4A1 (NM_001845.6)	GCTGTGGATCGGCTACTCTT	GGACGGCGTAGGCTTCTT
COL7A1 (NM_000094.4)	CCCACATCCATCCTCCTTT	CCCATCCAACTGGTAGCG
COL17A1 (NM_000494.4)	GAGGACGGAGTCAAACACG	CTTGAGCAAACGCTTAACAT
COL24A1 (NM_152890.7)	AATCTCAAGAAGGCTATCAC	ATGGCTCATTTGTCACTC
FN1 (NM_212482.4)	TGTTATGGAGGAAGCCGAGGTT	GCAGCGGTTTGCGATGGT
ELN (NM_000501.4)	CCCGCAGTTACCTTTCCG	GGCACTTTCCCAGGCTTCA
CXCL1 (NM_001511.4)	CCCCAAGAACATCCAAAGTG	GATGCAGGATTGAGGCAAG
CXCL5 (NM_002994.5)	TACAGACCACGCAAGGAGTT	TCTTCAGGGAGGCTACCAC
CXCL7 (NM_002704.3)	AGTGCGAGACCACTTCAT	ACTTTCCTCTTTGCCTTT
CXCL8 (NM_001354840.3)	TGGCAGCCTTCCTGATTT	ACTTCTCCACAACCCTCT
CXCL17 (NM_198477.3)	TGCTGCCACTAATGCTGA	TGGTGCCTTTGGTGTCTT
MMP1 (NM_002421.4)	GGCTGAAAGTGACTGGGAAAC	GGCAAATCTGGCGTGTAA
IL-1β (NM_000576.3)	ATGGCTTATTACAGTGGC	TAGTGGTGGTCGGAGATT
PGE2 (U19487.1)	CGAGGTGTATGTATGAGTGT	AGTGGGTAAGTATGTAGTGC
GAPDH (NM_001289746.2)	CTCATGCGCTGTGTGGAA	GAAAATGGGAAACTGGCT
TGFB1-3′UTR (NM_000660.7)	GGACTCTGATAACACCCATTT	ATTACAGGCGTGAGCCAC
COL1A1-3′UTR (NM_000088.4)	CTCAGACTGCCAAAGAAGC	GCACAAGGGATTGACACG
CXCL8-3′UTR (NM_001354840.3)	TGGGTTTGCTAGAATGTG	GTGAGGTAAGATGGTGGC
COL4A1-3′UTR (NM_001845.6)	TCTGCATCCTGGCTTGAA	TCCGAATCTGCCCTCCTG

## Data Availability

All the data used to support the findings of this study are available from the corresponding author upon reasonable request.
